# Diseases of the Stomach, Liver, and Intestines

**Published:** 1903-08-22

**Authors:** 


### Diseases of the Stomach, Liver, and Intestines.

*(Concluded from page 349.)*

**Pancreas.**—Deaver[12] gives an exhaustive account of the anatomy, physiology and pathology of the pancreas, classifies its diseases and quotes illustrative cases. The chief lesions are pancreatitis, acute, subacute, and chronic, lithiasis, cysts, and neoplasms. Injuries are rare. Details of surgical measures are given, and a note added on *x*-ray diagnosis by Wilbert. The importance of biliary calculi in the ætiology of pancreatitis is insisted upon and operation advised if the general condition permits. Where gangrene and suppuration have occurred it is imperative. The diagnosis of chronic pancreatitis from malignant disease is difficult and rests in the extreme cachexia, absence of pain, extreme jaundice, ascites, and enlargement of the liver and gall-bladder found in the latter. Operation is only practicable in the former. The majority of epigastric cysts are pancreatic; six varieties are mentioned. Non-malignant growths and syphilis are very rare. Highett[13] describes a case of malignant disease of the pancreas which he thinks might have been correctly diagnosed by a process of exclusion, had he remembered Dr. Roberts' warning—"Many seem to forget that there is such an organ." Hardin[14] considers chronic pancreatitis a not uncommon and usually infective condition. Failure in pancreatic secretion is an aid to its recognition, and causes much muscle fibre to reappear in the stools and no carbolic acid reaction in urine after administration of salol. The treatment is purely surgical.

**Peritoneum.**—A case of chyliform ascites has been described by Corney and McKibben[15] characterised ised by symptoms of obstruction to the thoracic duct complicated by attacks of cellulitis. The condition was caused by the pressure of an old tuberculous focus. Actual chyle was not demonstrated in the peritoneum. Veit[16] considers that tuberculous peritonitis is always secondary. He advises laparotomy except where tubercle is general. Rotch[17] analysed 69 cases of laparotomy, and states that tuberculosis of mesenetric glands is no contra-indication. The after history of 13 cases showed that 10 were alive and well at periods ranging from three months to nine years after. Symptoms are indefinite, no reliance can be placed on the state of the bowels or blood. Pyrexia is irregular. There is usually no tenderness. Bellamy[18] describes a case where symptoms of tuberculous peritonitis existed, but cleared up under treatment. There was a strong family history of tuberculosis.

**Dietetics.**—Hutchison[19] warns us against assuming that man's diet under civilised conditions should be regulated by what is "natural" to him in the savage state. Food must consist of proteid, fat and carbohydrate; it must supply about 3,000 calories daily and sufficiently replace body waste. How much is necessary for the latter process is uncertain. Patent condensed foods are as inadvisable as they are unsatisfying. Many diseases cannot be influenced at all by diet; those which can are: (1) Diseases of the digestive organs, (2) diseases of metabolism, (3) diseases of the excretory organs. In all cases personal idiosyncrasies must be considered, the general strength must not be reduced for the sake of one organ, and no changes should be abruptly made. (*a*) Digestive organs: The dyspepsia caused by organic disease requires food easily soluble and of small bulk. Chemical composition is not so important as physical form. In "functional" dyspepsia elaborate dietaries are a mistake. (*b*) Metabolic diseases: Fevers require carbohydrates rather than fats; liberality in diet should be proportional to the chronicity of the condition, unless there is a local lesion as in enteric. Obesity is often uninfluenced by diet, but in some cases a proteid diet does good. Alcohol is always harmful, and liquids need only be restricted to decrease a tendency to over-eating. In malnutrition proteids are specially valuable; in diabetes, fats; in gout, uric-acid-forming foods should be avoided, such as flesh, meat extractives, and leguminosæ. Endogenous uric acid is formed irrespective of diet. (*c*) Excretory organs: In acute renal disease milk is indicated, possibly as an antiseptic. In chronic cases a mean must be struck between over-stimulation and semi-starvation. White meats contain as much purin bodies as red, and restriction of water helps to remove dropsy, and does not interfere with due elimination of waste. Herschell [20] contrasts the principles of diet in organic and functional disease of the stomach. In hyperchlorhydria the differences are mainly in the mode of administering food according to the causation of the disease. In subacidity carbohydrates should be increased. In motor disturbances, if the stomach is not irritable, stimulating foods should be given, but if irritable, bland foods. In all cases water should be reduced, and taken about one hour before food. Pyorrhœa alveolaris may induce chronic gastric disturbances. Williams [21] condemns alcohol and stimulating foods in nervous diseases. The form of alcohol should be that of the patient's country. Young [22] records a severe case of sprue in which, after 12 months' illness, milk diet restrained the diarrhœa. Small additions of other substances were risky. Finally strawberries, in large quantities, seem to have had a marked effect in restoring strength and rendering other foods digestible. The dietetic value of oysters [23] has been investigated. Though an expensive food, they are not much inferior to meat as regards the proportion of foodstuffs they contain. Expensive oysters are generally heavier and less watery though of the same composition as cheap ones. Luff [24] advocates more careful attention to diet in English health resorts. In gout he would not exclude meat, believing that the disease is not due to exogenous uric acid. Simple diet and avoidance of fermentable carbohydrates is advocated ; their place should be taken by fat, and alcohol avoided. Lean meat and water alone, though sometimes useful, is contra-indicated in advanced renal and uncompensated heart disease. Liberal diet should be given in rheumatoid arthritis.

**Therapeutics.**—Robertson [25] has investigated the action of sialogogues of which he finds pyrethrum, colchicum, ginger, horseradish, and black pepper the most efficient. The saliva thus excited is physiologically normal. Their use is advised in hyperchlorhydria to increase the salivary digestion of starch and neutralise the acid, and in gastric feebleness where saliva excites a further flow of gastric juice. Reginald Harrison [26] notes a peculiar idiosyncrasy against boric acid in himself and others, and advises boracite as a substitute in cystitis. Sohlern [27] describes the treatment of spastic intestinal dyspepsia found in neurasthenic individuals and characterised by pain, constipation, and a hard, band-like condition of the intestinal walls. Rest in bed, bland nutritious diet, warm abdominal applications alternating with cold ones, are advised. Belladonna and bromides are useful with oil and chloral enemata, but drastic purges, electricity, and massage are to be avoided. Lott [28] states that most secret cures for the drug habits consist in intoxicating the patient for 48 hours with duboisine, atropine, strychnine, hyoscyamine, pilocarpine or the hydrobromate of hyoscine, which last is best. Large aggregate doses are given, and the patient, who becomes delirious, requires careful watching. After this pilocarpine is given to eliminate the hyoscine. The third and most difficult stage consists in treating the unpleasant after conditions ; the craving for the drug ceases after the first stage, but will return unless the whole process is carried out, and the patient restored to natural health.

**Alcohol.**—French [29] medical men have been debating the action of alcohol as a food, and the general result favours its use in strict moderation. Duclaux, Roux, and Richet believe it is a food, while Metchnikoff considers it a poison. Berthelot thinks it valuable as a drug. Brouardel and Héricourt doubt whether alcohol is really a food, while Magnan says that at any rate it is not a good one. Bernheim, Lancereaux, Landouzy and Bourneville are generally inclined to allow its value as a drug and its harmlessness in moderation as a beverage. Garnier attributes 70 per cent. of crime in France to alcohol. In England Sims Woodhead [30] considers alcohol a cell-poison tending invariably to produce degeneration in protoplasm. As a usually secondary result it causes connective tissue formation. It also checks the elimination of waste products, prevents the acquisition of immunity, and is of little value as a food. Glover [31] favours extreme moderation in its use. It is unnecessary in childhood and should be guardedly taken in old age. Statistics in Edinburgh and Glasgow show that it is largely responsible for increase in pauper insanity. Reports on the physical deterioration of recruits show both its direct and indirect effects—money spent on alcohol by parents should have been spent on food for their children. It predisposes to phthisis, and other drugs are of more value in treating carbuncle and fevers. Total abstinence is not possible in cold countries, and alcohol has still a definite place in medicine. Morison [32] considers it of great value in cardiac failure, shock and exhaustion from any cause, and in health to aid digestion and relieve vascular tension. Anderson [33] considers it really valuable in malnutrition and exhaustion, harmless in moderation as a beverage, and useful as a generator of energy.

[12] Amer. Jour. Med. Sci., Feb. 1903, p. 187. [13] Brit. Med. Jour., Jan. 10, 1903, p. 75. [14] Med. Rec., Feb. 28, 1903, p. 348. [15] Lancet, March 21, 1903, p. 825. [16] Med. Rec., Jan. 31, 1903, p. 191. [17] Ibid., Jan. 17, 1903, p. 109. [18] Lancet, May 2, 1903, p. 1230. [19] Ibid., April 25, 1903, p. 1154. [20] Ibid., p. 1171. [21] Ibid. [22] Ibid., March 28, 1903, p. 873. [23] Ibid., Jan. 3, 1903, p. 71. [24] Ibid., May 2, 1903, p. 1224. [25] Ibid., June 13, 1903, p. 1673. [26] Ibid., March 21, 1903, p. 836. [27] Brit. Med. Jour. Epitome, Feb. 21, 1903. [28] Ibid., March 7, 1903. [29] Brit. Med. Jour., March 14, 1903, p. 622. [30] Lancet, June 13, 1903, p. 1670. [31] Ibid. [32] Ibid. [33] Ibid.

